# Debate Regarding Oseltamivir Use for Seasonal and Pandemic Influenza

**DOI:** 10.3201/eid2206.151037

**Published:** 2016-06

**Authors:** Aeron C. Hurt, Heath Kelly

**Affiliations:** World Health Organization Collaborating Centre for Reference and Research on Influenza, The Peter Doherty Institute for Infection and Immunity, Melbourne, Victoria, Australia (A.C. Hurt);; University of Melbourne, Parkville, Victoria, Australia (A.C. Hurt);; The Peter Doherty Institute for Infection and Immunity, Melbourne (H. Kelly);; Australian National University, Canberra, Australian Capital Territory, Australia (H. Kelly)

**Keywords:** oseltamivir, influenza, antiviral agent, treatment, stockpiles, stockpiling, viruses, respiratory diseases, pandemic

## Abstract

A debate about the market-leading influenza antiviral medication, oseltamivir, which initially focused on treatment for generally mild illness, has been expanded to question the wisdom of stockpiling for use in future influenza pandemics. Although randomized controlled trial evidence confirms that oseltamivir will reduce symptom duration by 17–25 hours among otherwise healthy adolescents and adults with community-managed disease, no randomized controlled trials have examined the effectiveness of oseltamivir against more serious outcomes. Observational studies, although criticized on methodologic grounds, suggest that oseltamivir given early can reduce the risk for death by half among persons hospitalized with confirmed infection caused by influenza A(H1N1)pdm09 and influenza A(H5N1) viruses. However, available randomized controlled trial data may not be able to capture the effect of oseltamivir use among hospitalized patients with severe disease. We assert that data on outpatients with relatively mild disease should not form the basis for policies on the management of more severe disease.

## Abstract 2

Data on outpatients with relatively mild disease should not form the basis for policies on the management of more severe disease.

A lively, and sometimes heated, debate has recently been conducted in the popular press (*1*) and medical literature (*2**–**4*) about the effectiveness of oseltamivir, its usefulness in treating seasonal influenza, and the need for it to be stockpiled for use in a future influenza pandemic. Oseltamivir, manufactured by F. Hoffmann-La Roche (Roche) (Indianapolis, IN, USA), is the market leader of the neuraminidase inhibitors (NAI), the first class of antiviral drugs designed specifically to treat influenza.

Oseltamivir came on the market in many countries in 2000 after clinical studies had been conducted among influenza virus–infected patients with uncomplicated illness. Trial data from outpatient studies have been summarized in a recent meta-analysis of individual patient data, including published and unpublished studies, which confirms that oseltamivir will reduce the duration of symptomatic laboratory-confirmed influenza in otherwise healthy adults from 5 days to 4 days (*2*), a result consistent with those of a previous systematic review by the Cochrane group (*3*).

After the drug’s market release, oseltamivir use for treatment of seasonal influenza was modest in most countries, except for Japan, where widespread use of the drug was adopted. However, governments began to consider antiviral drug administration as a key component of their planned pandemic response after human infections with avian influenza A(H5N1) virus increased in 2003 and were associated with a case-fatality risk of >50% (*5*). Suitable vaccines would not be available at the start of a pandemic; thus, use of antiviral agents was seen as a critical part of a pandemic response.

Because the mode of administration for oseltamivir was simpler (oral) than that for zanamivir (inhalation) and because the systemic effect of oseltamivir was expected to be appropriate for treatment of highly pathogenic viruses, oseltamivir was suddenly in high demand, apparently driven by warnings from Roche that preemptive stockpiling was the only way that governments could be assured of drug availability (*6*). Since 2005, governments of middle-income and high-income countries around the world have spent billions of dollars (estimated) stockpiling oseltamivir (*7*). However, by November 2015, the influenza A(H5N1) virus that initiated stockpiling had caused only 844 human cases of infection and 449 deaths (case-fatality risk 53.2%) across 16 countries worldwide, with only 7 countries reporting >10 cases (*8*).

The first pandemic of the 21st century occurred unexpectedly in 2009 after the global spread of a novel virus—influenza A(H1N1)pdm09—of swine (rather than avian) origin. In response, many countries activated their stockpiles of antiviral agents or accessed existing community supplies. This was the first time that specific antiviral drugs were available in a pandemic. In the United States during 2009, 8.7 million oseltamivir prescriptions (28.4 prescriptions/1,000 persons) were dispensed from community pharmacies, not from the stockpile, at a cost of US $905 million (*9*).

Although the number of deaths due to the 2009 pandemic was lower than initially anticipated, a unique opportunity was provided to review the effectiveness of oseltamivir in the pandemic setting and to determine the benefit of oseltamivir for patients who were hospitalized with confirmed influenza A(H1N1)pdm09 virus infection. Such observational data were valuable to ascertain the effect of oseltamivir in severely ill or hospitalized patients given the continued absence of data from placebo-controlled, randomized controlled trials.

Questioning whether oseltamivir is useful for treating serious illness and whether it should be stockpiled has extended the debate on the effectiveness of oseltamivir in the community. We believe that these issues should be considered separately.

## Oseltamivir Treatment of Seasonal Influenza 

After partially successful efforts to retrieve unpublished data from Roche (*10*,*11*), the Cochrane group conducted a meta-analysis of the effectiveness of oseltamivir in treating uncomplicated community-acquired influenza. Their findings led the group to conclude that oseltamivir had no specific antiviral effect, even though the drug had been specifically designed to achieve exactly that (*3*). The Cochrane systematic review, which focused only on an intention-to-treat analysis, confirmed that oseltamivir reduced symptom duration in the intention-to-treat group by <24 hours. Earlier, the Cochrane group had noted, “We are unsure of the generalizability of our conclusions from seasonal to pandemic or avian influenza” (*12*).

As noted previously, a subsequent meta-analysis (funded by an unrestricted grant from Roche) also confirmed a ≈1-day reduction in symptoms among adults and adolescents who had laboratory-confirmed influenza and were treated within 48 hours of symptom onset (*2*). This analysis included outcomes of both intention-to-treat and intention-to-treat-infected groups. Benefit was found for the intention-to-treat-infected group, but no benefit was found for patients with influenza-like illness who did not have laboratory-confirmed influenza (the intention-to-treat but not infected group) (*2*). Given that the benefit of oseltamivir was confined to symptomatic patients with laboratory-confirmed infection, the authors concluded that the effect of oseltamivir was due to its effect on the influenza virus, rather than a nonspecific antiviral effect, as had been suggested by the Cochrane group (*3*).

Secondary analyses from the Roche-sponsored meta-analysis suggested the following: a 63% (95% CI 19%–83%) decreased risk in hospitalization for any cause, based on 9/1,591 (0.6%) oseltamivir treated vs 22/1,302 (1.7%) placebo-treated patients; and a 44% (95% CI 25%–58%) decreased risk of antibiotic prescription for lower respiratory disease in patients with laboratory-confirmed influenza, based on 65/1,544 (4.2%) oseltamivir-treated vs 110/1,263 (8.7%) placebo-treated patients. However, hospital admissions were all cause and not confined to those that may have been associated with influenza infection; also, no formal diagnostic criteria existed for lower respiratory tract infection (*2*,*13*). We consider these secondary analyses less convincing than the analyses of primary endpoints. The latter were largely in agreement with those of the Cochrane group. Yet, despite this agreement and the arm’s length funding mechanism, the Roche-sponsored meta-analysis has been criticized as being influenced by the manufacturer (*14*).

## Oseltamivir Treatment of Severe Influenza 

Although necessary to consider oseltamivir’s effect on more serious infections, no randomized controlled trials exist that can be included in a meta-analysis. The Cochrane group chooses only to conduct meta-analyses of randomized controlled trials, which are generally accepted to be the highest level of evidence. Thus, the Cochrane group could not review severe outcomes of laboratory-confirmed influenza. Evidence is instead derived from observational studies on the use of oseltamivir to treat complications of influenza virus infection, as in hospitalized patients or in those who died. These studies are subject to uncontrolled bias. For instance, sicker patients may be more (or less) likely to be treated, thus attenuating (or exaggerating) the effect of the intervention. Also, a serious outcome may occur soon after the treatment was initiated in a severely ill patient, so that the treatment has not had a chance to succeed. Similarly, patients who receive early treatment are more likely to benefit from treatment than patients who receive late treatment. To minimize bias, researchers conducting observational studies have attempted to adjust for time from disease onset to treatment and time from treatment to outcome. Some observational studies have also adjusted for propensity to be treated as well as patient coexisting conditions and disease severity, which may affect treatment decisions and outcomes.

Observational studies that enrolled adults have often used death as an outcome, given its ease of definition. However, many observational studies fail to control completely for potential biases, including time biases. Among studies that have attempted to control for these biases, a decreased risk for death after oseltamivir treatment has been reported, and early treatment appears to be critical (*4*,*15*,*16*). For instance, in a retrospective cohort study from Israel of 449 patients hospitalized with influenza A(H1N1)pdm09 infection, all patients were treated with oseltamivir, and 189 (42%) were treated within 48 hours. This observational study controlled for propensity to treat and patient coexisting conditions and demonstrated the odds of death increased by 2.2 times (95% CI 1.4–3.5 times) if treatment was started late (>48 hours after symptom onset) (*16*).

In an attempt to overcome the criticisms of design and analysis of the observational studies, Roche chose to fund a patient level meta-analysis of individual data from 78 different observational studies, which included >29,000 patients. By adjusting for time, propensity to treat, and patient coexisting conditions, and comparing the effect of treatment with no treatment in patients infected with influenza A(H1N1)pdm09, researchers found that the odds of death were reported as 0.50 (95% CI 0.37–0.67) for adults whose treatment was initiated within 48 hours, compared with the odds of death for untreated adults (*4*). However, in a series of exchanges published in the British Medical Journal and The Lancet Respiratory Medicine, even this carefully designed study has been criticized on methodologic grounds (*17*–*20*). A more recent meta-analysis of individual patient data from the same group of investigators examined the potential effect of oseltamivir on influenza-related pneumonia among >20,000 patients with laboratory-confirmed influenza A(H1N1)pdm09 (*21*). However, this study has many of the predictable methodologic problems associated with retrospective reviews and does not add to the evidence base. There is scant other evidence for the benefit of oseltamivir on reducing the risk for death to help resolve residual uncertainty. The few potentially informative observational studies report on human infection with avian influenza strains (*22*) and seasonal influenza (*15*,*23*), including an unpublished review sponsored by Roche (*24*,*25*).

In a review of individual patient data for 308 patients from observational studies conducted in 12 countries, based on data from a patient register funded by Roche, oseltamivir treatment was reported to decrease the risk for death from influenza A(H5N1) virus by 49% (95% CI 23%–66%). The analysis of risk for death was restricted to 258 patients from 7 countries; mean values were substituted for missing data (*22*).

Two prospective observational studies of hospitalized patients with laboratory-confirmed seasonal influenza have shown oseltamivir treatment decreases the risk for death. In a prospective observational cohort study from Hong Kong, which enrolled 754, mostly elderly, hospitalized patients with co-existing conditions during 2007–2008, oseltamivir treatment was associated with a reduced risk for death (adjusted hazard ratio 0.27, 95% CI 0.13–0.55; p<0.001), with a further reduction associated with earlier treatment (*15*). A small prospective observational study of patients hospitalized with laboratory-confirmed influenza in the 2005–06 season in Ontario, Canada, found the adjusted odds ratio of death among oseltamivir-treated patients was 0.21 (95% CI 0.06–0.80; p = 0.03), based on 22 (10%) of 219 deaths in the untreated group compared with 4 of 103 deaths in the treated group (*23*).

Also supporting the conclusion that oseltamivir use has a beneficial effect on reducing the risk for death were findings from a large review from the Ingenix Research Data Mart (*24*), apparently sponsored by Roche. We have only been able to find an abstract of the study with an associated commentary (*25*). The observational study found that oseltamivir use decreased the risk for death in patients of all ages with influenza (1 death/39,202 patients) compared with untreated patients (56 deaths/136,799 patients; p = 0.02). However, the commentary raised several issues related to study design, which could not be resolved without further detail (*25*).

We have not been able to find an analysis of oseltamivir effectiveness for treating infections with avian influenza A(H7N9) virus in China, a virus that, since its emergence in 2013, has caused a greater number of annual cases and deaths than influenza A(H5N1) virus. Such a study might also contribute to the evidence base.

## Oseltamivir Policies for Seasonal and Pandemic Influenza

The evidence from randomized controlled trials is clear that oseltamivir treatment decreases the duration of symptoms by up to 1 day in adolescent and adult patients with laboratory-confirmed seasonal influenza whose infections are able to be managed in the community. Oseltamivir provides no benefit to patients who have influenza-like illness not caused by influenza virus (*2*). Reviews of observational data regarding patients hospitalized with influenza A(H1N1)pdm09 or influenza A(H5N1) infections found that risk for death is cut in half if treatment is initiated within 48 hours of symptom onset (*4*,*22*). Small prepandemic observational studies, although they generally have controlled less for potential biases, also support the conclusion that risk for death is decreased with oseltamivir treatment (*15*,*23*).

These 2 lines of evidence may appear inconsistent. How would an intervention that has a modest effect on symptom duration in patients whose uncomplicated influenza was treated after a visit to a general practitioner be able to cut in half the risk for death among hospitalized patients?

It should not be surprising that treatment for uncomplicated influenza will only produce a modest effect because influenza virus replication precedes symptoms by 1–2 days. This means that the viral load in the patient may have peaked by the time the patient begins antiviral treatment. Given that most antiviral drugs, including oseltamivir, act by reducing viral replication, the effect of treatment will therefore be minimal in otherwise healthy persons when immune responses are already reducing viral titers. It is therefore plausible that community-based randomized controlled trials are not capturing critical information about the mode of action of oseltamivir that is beneficial to severely ill patients.

It is possible that benefit to severely ill patients may be related to the increased duration of viral shedding and higher viral loads found in this group of patients (*26*). Elevated cytokine levels, sometimes referred to as a cytokine storm, have been detected for patients infected with highly pathogenic A(H5N1) virus (*27*) and for severely ill patients infected with influenza A(H1N1)pdm09 and seasonal influenza viruses (*28*,*29*). A randomized controlled trial study of 117 healthy adults experimentally infected with seasonal influenza virus A(H1N1) reported that oseltamivir treatment significantly reduced interleukin-6, interferon-γ, and tumor necrosis factor-α cytokine responses in patients compared with responses in placebo-treated patients (*30*). Although these results do not clarify whether the decreased cytokine response was the result of effective viral treatment or a (postulated) immune modulatory effect of oseltamivir, ferret studies conducted in our laboratory suggest that oseltamivir treatment consistently reduces peak temperatures and improves ferret activity/wellness but often does so in the absence of any significant effect on viral load (*31*). The difference in outcome for severely infected patients treated with oseltamivir may relate to decreasing the adverse outcome associated with a cytokine storm, which would not be expected in patients with mild disease.

## Implications for Stockpiling

Data from the randomized controlled trials of patients with mild influenza and the observational data from severely ill patients demonstrate the clear clinical benefits of initiating treatment as early as possible after infection (*32*). NAI use in Japan is so widespread that almost every confirmed influenza case is treated, which is likely to have led to the extensive and rapid delivery of the drugs in the 2009 pandemic. For instance, in a study of Japanese children hospitalized with influenza A(H1N1)pdm09 virus infections, >98% (984/1,000) were treated with an NAI, and for those for whom the treatment time was recorded, 89% received NAIs within 48 hours and 70% within 24 hours. Only 1% of the hospitalized children ultimately required mechanical ventilation, and 1 death was recorded (*33*).

Similar ecologic data were observed among pregnant women in Japan, a group of patients at increased risk for hospitalization and death when infected with influenza A(H1N1)pdm09. Pregnant Japanese women were treated prophylactically after close contact with an infected person, and if infected and hospitalized, >90% were given NAIs within 48 hours of symptom onset. In comparison to the high mortality rates among pregnant women in many countries around the world (*34*), no maternal deaths occurred in Japan during the pandemic (*35*).

Ecologic data from Japan, although regarded as the weakest form of epidemiologic evidence, suggest that rapid access to stockpiled NAIs in a pandemic is necessary to achieve the greatest benefit from their use. Rapid access during the 2009 pandemic in Japan was possible because rapid access represented routine care for seasonal influenza. In other countries, the 2009 pandemic confirmed that centralized stockpiles did not facilitate rapid distribution (*36*) and that decentralized stockpiles would be more efficient. Stockpiles in hospitals, for example, would facilitate rapid treatment of ill patients in a pandemic but might also allow the periodic use of some material for the treatment of interpandemic seasonal influenza to avoid wastage due to an expiring stockpile (*36*).

## The Way Forward

There is general agreement derived from randomized controlled trials about the modest effectiveness of oseltamivir against relatively mild illness in otherwise healthy persons, but several lines of evidence from observational studies suggest that oseltamivir decreases the risk for death (*37*). However, within the next 5–10 years, we do not expect to see clarifying new evidence from trials of patients recruited from the community who have an endpoint of severe influenza. Severe outcomes from influenza are uncommon, as shown in the meta-analyses of community trial data, and randomized controlled trials that recruit healthy persons would require extremely large patient numbers and need to be conducted over multiple seasons to account for potentially different outcomes by influenza type and sub-type.

On the other hand, a randomized controlled trial that recruited only patients with severe influenza, although feasible from a design perspective, could not ethically evaluate active treatment versus placebo treatment because oseltamivir treatment is the standard of care for patients with severe influenza virus infections. In a study that overcame the ethical issue, a randomized controlled trial of patients with severe influenza that examined single-dose versus double-dose oseltamivir found no difference in outcomes between the 2 treatment arms (*38*). We do not anticipate that this trial would be repeated.

Existing observational evidence on the benefits of oseltamivir for the treatment of influenza in hospitalized patients, including assessing the risk for death, accrues from ecologic data from Japan, weak secondary analyses from randomized controlled trials of laboratory-confirmed influenza initially managed in the community, and the systematic reviews and analyses of observational studies of patients with confirmed infections caused by influenza A(H1N1)pdm09 and influenza A(H5N1) viruses. Methodologically less robust, small observational studies conducted before the pandemic support the finding that oseltamivir decreases the risk for death. However, well-designed prospective observational studies may provide the most informative data in the next 5–10 years.

We reached several conclusions regarding the use of oseltamivir and the considerations that will be necessary for future studies (Table). Nguyen-Van-Tam et al. recently outlined a list of covariates that would need to be collected to help strengthen the evidence that oseltamivir treatment benefits hospitalized patients with influenza (*39*). These include standardized data on illness onset and progression, comorbid conditions, disease severity, treatment, duration of hospital stay, the need for critical care, and influenza-related mortality. In addition, a review by the UK Academy of Medical Sciences, sponsored by the Wellcome Trust, has offered a range of approaches to potential future studies and called specifically for “pragmatic or adaptive [randomized controlled trial] designs” of neuraminidase inhibitors in hospitalized patients (*37*). Our conclusions are in broad agreement with those of this report.

**Table:** Conclusions of study evaluating the use of oseltamivir for seasonal and pandemic influenza and wisdom of stockpiling*

Summary conclusions
1. Although debate continues, there is general agreement from meta-analyses of RCTs that oseltamivir reduces symptoms in healthy adults and adolescents with influenza by up to 1 day. There is disagreement on the mechanism. On 1 side of the debate, the Cochrane group maintains that there is a nonspecific effect of oseltamivir, whereas, on the other side, investigators sponsored by Roche maintain that oseltamivir has a specific anti–influenza virus effect.
2. There have been no RCTs that can be meta-analyzed to summarize the effect of oseltamivir on severe outcomes of influenza virus infection. Evidence derived from observational studies of serious outcomes consistently suggests that oseltamivir reduces the risk for death in severely ill patients with documented influenza infection.
3. The apparent discrepancy between a modest drug effect for healthy persons and a substantial effect on number of deaths remains unexplained. Currently, oseltamivir is the only licensed drug available for all ages.
4. Based on available evidence, oseltamivir should be used for treatment of hospitalized patients with laboratory-confirmed seasonal influenza and stockpiled for the treatment of patients with severe laboratory-confirmed pandemic influenza, whether hospitalized or not. These stockpiles should be widely distributed to facilitate rapid use when needed.
5. Without a mechanism for rapid distribution of the drug in an emergency, any potential benefit of such large-scale stockpiling will not be realized. Rapid distribution in an emergency is only likely if a mechanism exists for routine rapid distribution. In countries where such a mechanism does not exist, we see no place for stockpiling oseltamivir for widespread community use during a pandemic.
6. It is unlikely that conventional RCT-level evidence to support antiviral treatment of severe laboratory-confirmed influenza in hospitalized patients will appear within the next decade due to the ethical constraints of evaluating oseltamivir vs placebo, when oseltamivir is the current standard of care for the treatment of severe influenza infection. New studies should be pragmatic trials or high-quality prospective multisite observational studies and employ methods to minimize bias to the greatest extent possible.
7. Studies designed for assessing interventions for seasonal influenza should be readily adaptable to studies of pandemic influenza on very short notice. Because of the ethical and design constraints of RCTs, prospective observational studies are more feasible than RCTs in an emergency response situation. In addition to data on outcome, such as risk of ICU admission and death among adults, or length of stay among children, these observational studies should also record time from disease onset to treatment and time from treatment to outcome to minimize bias. Sequential data on markers of immune function in at least a subset of recruited patients would also be valuable.

*RCT, randomized controlled trial; ICU, intensive care unit.

The details of study design may be best accomplished by an experienced group of international researchers, focusing on standardized recruitment procedures and covariate and outcome definitions with a clearly defined analysis plan designed to minimize bias, as far as possible. Funding for such studies could come from the public or private sectors, with prior safeguards on perceptions of conflicts of interest. The studies should be adaptable to evaluate new antiviral medications, including intravenous forms of the NAIs, newly licensed non-NAI antiviral agents that are currently in late-phase clinical trials, or adjunctive therapies and immunomodulatory agents. Combinations of novel drugs with existing NAIs will likely be a useful approach and will require evaluation. Plans for ensuring broad availability and regulatory approval for emergency use of unlicensed antiviral agents will need to be established on very short notice. If pragmatic trials or high-quality prospective observational studies are completed and published over the next decade, an improved evidence base may help clinicians and public health planners decide on the most appropriate use of oseltamivir and potential new influenza antiviral agents for patients with severe infections caused by seasonal or pandemic influenza.
